# Freud and collocation: a psychodynamic interpretation of ‘*make*’ and ‘*do*’ in English

**DOI:** 10.3389/fpsyg.2026.1741902

**Published:** 2026-04-01

**Authors:** Rasheed Al-Jarrah, Muath Algazo, Musa Alzghoul, Bilal Alsharif

**Affiliations:** 1 Department of English Language & Literature, Yarmouk University, Irbid, Jordan; 2 Department of English Language & Literature, Al-Zaytoonah University of Jordan, Amman, Jordan; 3 Department of English Language & Literature, Mutah University, Alkarak, Jordan

**Keywords:** conceptual semantics, Freud’s structural model, ‘*make*’ vs. ‘*do*’, psycho-dynamic linguistics, psychological collocation

## Abstract

This study explores the relationship between everyday English verb choice and Freud’s structural theory of personality. Using a qualitative conceptual analysis, the research examines approximately 100 collocational expressions involving the verbs ‘*make’* and ‘*do’*, drawn from major learner dictionaries and the British National Corpus (BNC). Expressions were selected based on frequency, stability, and relative context-independence. The data were classified according to semantic orientation and interpreted through Freud’s id–ego–superego framework to investigate whether patterns of verb choice reflect underlying psychological structures. The findings suggest that ‘*make’* tends to align with instinctive or desire-driven actions, whereas ‘*do’* is associated with regulatory and task-oriented behavior. The study argues that linguistic collocation may encode traces of psychodynamic organization and offers theoretical implications for language teaching and translation, while acknowledging that these applications require future empirical validation.

## Introduction

Freud’s structural theory of personality has been highly influential in psychology, cultural studies, and critical theory (Busch and Delgado, 2024; Freud, 1923). While the model has been debated with respect to empirical validation, it continues to function as a powerful interpretive framework for analyzing human motivation, symbolic behavior, and meaning-making. Contemporary psychodynamic approaches draw selectively on its conceptual foundations to explore the complexity of mental life. In the present study, Freud’s model is employed not as a testable empirical claim about the brain, but as a theoretical lens for interpreting patterns in linguistic behavior. Central to Freud’s theory is the interaction between the conscious and the unconscious mind; hence, Freud (1923) argues that an individual’s behavior is driven by both. The structural theory underlies the assumption that whereas one’s conscious mind regulates a small portion of human behavior, much of it is driven by desires, feelings, and thoughts that fall beyond one’s conscious awareness. In this model, three intertwined personality components that influence human behavior interact: the id, ego, and superego. Whilst each plays its unique role, they together create a highly complex landscape of internal conflict. The model has been exploited widely and used extensively in psychology and cultural discourses.

Interestingly, although Freud’s theory was primarily articulated to explore the unconscious mind to understand human behavior, it has been drawn upon to explore human existence and origin (Freud, 1923; Ricœur, 1970). The theoretical premises of the proposal have been combined with the Darwinian views on human origin and evolution, part of which is the mystery of Man’s linguistic behavior (Christiansen and Chater, 2008; Pinker, 2007). The debate has been focused on whether words come from a divine source, are naturally symbolic, socially constructed or genetically grounded (Aitchison, 2000; Bickerton, 1990; Eco, 1997).

Several theories, such as the innateness hypothesis (Chomsky, 1965), hold the view that speech is genetic mainly—humans are born with the faculty/knowledge to acquire language almost effortlessly. Language learnability strongly supports this view; hence, despite the poverty of the stimulus (insufficient input), children develop the skills to accurately acquire complex linguistic structures (huge output) early in life in a relatively short time (Chomsky, 1965; Pinker, 2007). Considering the idiosyncrasy of the input each is exposed to, native language acquisition is never an inductive process. Linguists adopting the innateness (sometimes called nativist) view have been trying to find out how the language faculty works as a mental process. The most difficult part of the investigation is the one that targets the kind of behavior that is unobservable. In this paper, we claim that there is a close link between language and Freud’s structural theory. We argue that the personality components (the id, ego, and superego) constrain the words people use in daily life such as verbs like ‘*make’* and ‘*do’*. Specifically, we propose that the ego drives the use of ‘*do’*, reflecting order and control, whilst the id drives the use of ‘*make’,* reflecting impulse and creation. Although Freud’s model remains a central framework for understanding human nature, it has not been widely applied to explain linguistic behavior. Although psychoanalytic perspectives have influenced areas such as discourse analysis, literary studies, and theories of the unconscious in language, their application to everyday lexical collocation within cognitive linguistics remains limited. Existing work has explored symbolic and interpretive dimensions of language, yet few studies have examined routine verb choice through a Freudian structural framework. The present study addresses this gap by focusing specifically on the verbs ‘*make’* and ‘*do’* as a site where linguistic patterning may intersect with psychodynamic organization.

Building on cognitive linguistic approaches to meaning, this study investigates whether Freud’s structural theory can function as an interpretive framework for understanding patterns of everyday verb choice. Focusing on collocational contrasts in English, such as ‘let’s *make* coffee’ versus ‘let’s *do* coffee’, the analysis explores how these routine expressions may be conceptually motivated and how they can be read through a psychodynamic lens.

## Linguistic behavior

Whether language is socially constructed or genetically grounded is part of the nature–nurture debate that prevails in most other fields of inquiry. As the debate is focused on how much of it is biology, it moves into the realm of psychology and cognitive sciences. Cognitive linguistics has emerged as an interdisciplinary field concerned with pulling together works in psychology and linguistics. Until the beginning of the second half of the past century, most psycholinguistic attempts had been couched within the frameworks of behavioral psychology theories, primarily advocated by Bloomfield and Skinner and held from the 1920s to the 1950s (Bloomfield, 1933; Skinner, 1957). Starting in the early sixties and continuing over the seventies of the last century, Chomsky promoted the view that linguistics is a branch of cognitive sciences, thereby advocating the shift from empiricism to mentalism (Chomsky, 1965, 1975; Gardner, 1985). Despite this shift toward mentalist explanations, psychoanalytic models have only occasionally been applied to the study of everyday linguistic structure. While research has explored language and the unconscious in discourse and symbolic analysis, their systematic application to routine lexical collocation remains limited. The present study builds on this intersection by examining how Freud’s framework can function as an interpretive lens for patterns of verb choice.

Cognitive semantics, a sub-branch of cognitive linguistics, has explored the contextual-conceptual nature of meaning (Abu Helal, 2023; Allwood and Gärdenfors, 1999; Croft and Cruse, 2004; Geeraerts, 2010; Lakoff, 1987). More recent developments in cognitive linguistics have expanded this framework through usage-based and constructionist models that emphasize frequency, embodiment, and probabilistic patterning in language (Bybee, 2010; Goldberg, 2006; Ellis, 2005). Empirical corpus-based approaches have further demonstrated how linguistic structure emerges from repeated usage and distributional regularities (Gries, 2013; Stefanowitsch and Gries, 2003). These perspectives reinforce the view that meaning is shaped by mental representation while grounding analysis in observable linguistic data. The present study aligns with this tradition by combining conceptual interpretation with attention to stable collocational patterns. The goal has always been to draw a line of demarcation between the components that are context-dependent (viz. pragmatic) vis-à-vis those that are entirely conceptual (viz. semantic) (Evans and Green, 2018; Croft and Cruse, 2004; Geeraerts, 2010; Langacker, 1987). Parts of the attempts aim to explain how language is computed in the mind and in the brain (Langacker, 1987). Linguistic works manifested to uncover cognitive structure are linked to neuropsychology, the branch of psychology whose main aim is to explore how cognition and behavior are orchestrated in the brain (Bechtel, 2001).

### Freud’s theory of personality as an interpretive model of mental processing

Freud’s structural theory of personality has been a historically influential framework in clinical psychology, psychoanalysis, and cultural theory for examining the relationship between personality and behavior. In this study, terms such as *mental processing*, *psychological tension*, and *practicality* are used in a theoretical psychodynamic sense rather than as neurocognitive measurements. They refer to conceptual models of internal conflict and decision-making proposed within Freud’s framework. The analysis does not claim direct access to measurable brain states; rather, these terms function as interpretive constructs for describing symbolic patterns in behavior and language.

According to Freud’s structural model, thoughts, emotions, and actions are shaped by the interaction of three components: the id, ego, and superego. The ego mediates the competing demands of the id and the superego. Whereas the id is oriented toward instinctual gratification, the superego represents internalized social and moral norms (Freud, 1923). Within this framework, the ego operates as a regulatory mechanism that negotiates tensions between desire and constraint. The resulting behavior reflects a compromise between these forces, which Freud conceptualized as an ongoing dynamic of internal balance.

### Context-independent collocational patterns

People often use words interchangeably in casual conversation despite the fact that they know that words embody distinct meanings and functions. Word choice is sometimes motivated on contextual grounds (e.g., thanks vs. thank you), but it is not always possible to make recourse to context to explain the subtle differences between synonymous terms. Knowing that each can alter meaning drastically, linguists have been tasked to figure out how people use words to communicate effectively. Part of the effort has been put into figuring out the cognitive processes underlying the use of synonyms. The task is principally tackled in two overlapping subfields: semantics and pragmatics. Traditionally, a decontextualized word/sentence falls within the borders of semantic analysis. Pragmatics, on the other hand, pays special attention to the different contextual forces (e.g., speaker, hearer, audience, setting, etc.) that influence meaning. The goal of any meaning-oriented investigation, though not explicitly stated, is twofold: (1) knowing the part of meaning that is conceptual (semantic), and (2) finding out the part that is contextual (pragmatic). Although many studies acknowledge the role of context in shaping meaning, the precise balance between contextual (pragmatic) and conceptual (semantic) factors is not always systematically addressed. Cruse (2000) proposes a scale of synonymity consisting of absolute synonymy, cognitive synonymy, and near-synonymy. In natural languages, absolute synonymy is impossible or non-existent. Hence, its existence violates the economy principle (Cruse, 2000; Murphy, 2003). The fact of the matter is that no two or more terms are interchangeable in all contexts. Therefore, only two main types of synonymity have been subject to investigation: cognitive synonymy and near-synonymy (Lyons, 1995). Whereas near-synonymy is context-dependent, cognitive synonymy is not. According to Murphy (2003), words that are cognitively synonymous are sense synonyms.

What is more puzzling to the linguists are those collocational patterns that cannot be explained by recourse to context. Usages that do not necessarily depend on time, place, interlocutors, etc., are characterized as logical, referential, denotative (or denotational), descriptive, propositional, and conceptual (Cruse, 2000; Lyons, 1995). An example is the use of ‘*do’* and ‘*make’* in expressions such as ‘*make coffee’* vs. ‘*do coffee’*; hence, each has its unique meaning that hardly alters due to who uses it, when, and where it is used. Such usage differences are exclusively conceptual. Words such as these are cognitively synonymous, i.e., the difference is context-independent (Stanojević, 2009). As the difference lies mainly in conceptual representation, linguists/semanticists need to resort to cognitive psychology, which studies mental processes, to find out why, for example, ‘*do coffee*’ is used as an actual proposal to get together whereas ‘*make coffee*’ is a causal event to achieve a household chore (Evans, 2009; Lakoff and Johnson, 1999; Langacker, 2008). Inherent in this line of thinking is the belief that finding the true meaning of the lexeme/word/expression obviates the need to claim that it is polysemous (i.e., having more than one meaning); hence, each term (irrespective of type) has only one basic meaning encompassing all its usages (Croft and Cruse, 2004; Bybee, 2010). If this were true, word meaning investigation would become a window into conceptual organization and mental representation rather than a literal map of brain structure.

### Beyond grammar: the meaning of ‘*make*’ and ‘*do*’

Although ‘*make’* and ‘*do’* are two of the most commonly used verbs in the English language, they are among the most frequently confusing for English learners in second language (L2) contexts. Hence, English grammar books often fail to clarify distinctions between the two cognitively synonymous terms. With many exceptions that do not align with the basic definitions, most textbooks, even the most reliable, draw the difference along these lines: whereas ‘*make’* refers to the creation of something, ‘*do’* is used for actions, tasks, etc. that do not necessarily produce a physical object (See, for example, Perfect English Grammar, n.d.; Murphy, 2019; Swan, 2005). This explanation is also widely used in online guides such as Grammarly, which states: ‘*Do’* is a versatile verb used for actions and tasks that are often routine or abstract, while ‘*make’* typically refers to the act of creation, bringing something new into existence (Grammarly, n.d.). Such a purported distinction can justify why we ‘*make cake’* and ‘do the dishes*’*, but surely fails to tell why we ‘make a decision*’* but ‘do a favour*’*. The lack of clear grammar explanations causes learners to memorize specific phrases rather than internalize a general rule, which, no doubt, is available in the mind of the native speaker who rarely, if ever, confuses the two verbs (Almegren, 2022; Nesselhauf, 2005; Lewis, 2000). The phenomenon of confusing verbs such as ‘*make’* and ‘*do’* is therefore only present in the grammar books, but never in the minds of native speakers. The difficulty relates, we reckon, to the grammarians’ inability to figure out what goes on inside the mind of the native speaker who uses the two verbs distinctly with the least possible mental processing effort (Bybee, 2010; Ellis, 2005).

Apparently, the phenomenon is not unique to English and may be observed in many other natural languages. For example, Spanish uses the verbs ‘*nacer*’ and ‘*crear*’ to differentiate between what can be ‘*done’* versus what can be ‘*made’*. The French verbs “faire” and “créer” are used along similar lines. In Arabic, the verbs “ya’mal” and “yaf’al” are notoriously challenging for non-native speakers, often presented to learners as fixed expressions that often require memorization (Anderson, 2025; Dajani et al., 2014; Mace, 1999). All in all, the phenomenon appears to present a significant challenge to grammarians who, we reckon, may have struggled to fully account for the rich complexity of language use as innately structured in the mind of the native speaker who uses it almost effortlessly, yet perhaps misrepresented in grammar textbooks which inadvertently contribute to confusion among learners.

Generally speaking, previous research on the difference between ‘*make’* and ‘*do’* has apparently underscored the complexity of the subject matter, thus failing to enhance any linguistic understanding and/or to help devise any pedagogical strategies that would foster the language learner’s linguistic proficiency. From our personal field experience, we can tell that whereas native speakers can easily figure out the correct usage with the least possible effort, non-native speakers alternatively push themselves to the limit to learn the expressions off by heart on a one-by-one basis. This tells quite straightforwardly that the difference between the two synonymous terms, and ultimately their various usages, is largely conceptual, i.e., has to do with what goes on inside the user’s mind. The question, therefore, is: what is it that makes the native English speaker use ‘*make’* and ‘*do’* in their various collocational patterns with the least processing mental effort, but the task is genuinely challenging to non-native speakers? What is even more interesting is: why is it difficult for the linguist/grammarian/language instructor (and above all the ordinary language user) to suggest a clear-cut difference between the two?

The claim we put forward here is that the complexity of suggesting a clear-cut distinction relates to the triggering force in each native user’s mind. Being conceptual, cognitive psychology is the field of inquiry that can lend a helping hand in solving the puzzle. Working toward this goal, Freud’s structural theory of personality can, we argue, be informative in telling us why English speakers, like native speakers of almost all languages, use two verbs to distinguish between two tasks (e.g., *make coffee* vs. *do coffee*). Freud’s framework is not proposed as a substitute for usage-based or cognitive models, which successfully account for frequency effects and distributional structure. Rather, it offers a complementary interpretive perspective concerned with symbolic motivation and internal conflict, dimensions that are not always foregrounded in formal or probabilistic accounts of language. The present study therefore employs Freud’s model as an additional theoretical lens for interpreting why certain collocational contrasts acquire persistent psychological resonance.

### Collocational patterns in cognitive psychology

Devoid of contextual influences, a word should be treated as a symbol for another word, which in turn is a symbol of another, ad infinitum (Harnad, 1990; Searle, 1980). Knowing the meaning requires, we reckon, breaking the continuum, by telling the root cause of the difference between, say, English ‘*make’* and ‘*do’*, Spanish *nacer* and *crear*, French *faire* and *créer*, and Arabic *ya’mal* and *yaf’al*. This requires understanding the cognitive processes language users engage in when using such highly synonymous terms. Understanding the complexities surrounding the verbs ‘*make’* and ‘*do’* reflects the deeper linguistic structure that surely merits a more comprehensive understanding of how the language faculty is wired in. In this research endeavor, we aim to show that the difference has to do with the component of the personality (i.e., the id, ego, or superego) that triggers the action: Whereas make-usages are collectively primal demands of the id, *do*-usages are sub-categorically calls of the super/ego. Accordingly, all actions are id-driven, ego-realized, or superego-motivated. Despite the dichotomy, the ego is the mediator whenever conflict arises.

### Research design

This study adopts a qualitative conceptual analysis grounded in cognitive linguistics and Freudian psychoanalytic theory. Here, qualitative conceptual analysis refers to an interpretive method that examines patterns of meaning through theoretical modeling rather than statistical measurement, focusing on recurring semantic structures and their conceptual interpretation (Croft and Cruse, 2004; Geeraerts, 2010; Evans and Green, 2018). The aim is not to establish measurable correlations but to explore conceptual relationships between linguistic patterns and psychological frameworks.

The study bridges linguistic theory, cognitive semantics, and psychodynamic psychology by proposing a model that links collocational tendencies to mental processes underlying the production and interpretation of language. Freud’s framework is employed as a complementary interpretive lens rather than a substitute for usage based or cognitive models. Usage-based approaches account for frequency and distributional regularities; however, the present framework addresses symbolic motivation and internal conflict that extend beyond purely probabilistic accounts of language.

The data consist of a purposive sample of approximately 100 collocational expressions involving the verbs ‘*make’* and ‘*do’*. Expressions were drawn from major learner dictionaries and the British National Corpus (BNC). Selection criteria included frequency of use, relative stability of expression, and minimal dependence on situational context. The goal was to examine expressions that function as routine linguistic patterns rather than isolated creative uses.

The analysis proceeded in three stages. First, expressions were grouped according to their dominant semantic orientation, such as creative, procedural, or regulatory functions. Second, these patterns were interpreted through Freud’s structural framework, mapping tendencies onto conceptual categories associated with the id, ego, and superego. Third, the results were examined within cognitive semantic theory to evaluate how collocational meaning reflects broader patterns of conceptual organization. This process is interpretive rather than predictive and is intended to generate theoretical insight rather than empirical generalization.

### Data selection

The data consist of a purposeful sample of collocational patterns and idiomatic expressions featuring ‘*make’* and ‘*do’* selected according to explicit criteria. Items were included if they are (a) listed as conventionalized collocations or idiomatic expressions in at least two major learner references (e.g., *Cambridge Advanced Learner’s Dictionary*, *Oxford Collocations Dictionary*), or (b) presented as stable usage patterns in pedagogical grammar sources (Swan, 2005; Murphy, 2019). No minimum frequency threshold was imposed, since the aim of the study is conceptual and interpretive rather than statistical. The BNC was consulted only for attested examples and contextual verification, not for quantitative analysis or frequency-based selection. The study therefore does not constitute a corpus-driven investigation; corpora function solely as a confirmatory reference to ensure authenticity of usage.

### Analytical framework

The analysis was conducted in three stages. First, collocations were classified based on their dominant semantic or functional orientation (creative/productive vs. procedural/ routine). Second, each expression was interpreted through Freud’s tripartite model: actions reflecting impulsive, creative, or instinct-driven tendencies were associated with the *id*; those involving regulation, rationalization, or mediation were attributed to the *ego*; and those expressing moral judgment or adherence to social ideals were aligned with the *superego*. Finally, these linguistic-psychological associations were examined through the lens of cognitive semantics, particularly the theory of conceptual motivation (Lakoff, 1987; Croft and Cruse, 2004), to reveal how meaning emerges from underlying mental schemas.

### Validation and reliability

The categorization of collocations was reviewed by two linguistics experts to assess its theoretical coherence and conceptual consistency. Specifically, the reviewers evaluated the plausibility of the interpretive framework and provided feedback to refine the category boundaries.

### Ethical considerations

As the study relies exclusively on secondary linguistic and theoretical data, no human participants were involved. Hence, formal ethical approval was not required. Nevertheless, all sources were cited correctly and used in accordance with academic integrity and intellectual property standards.

### Id-initiated actions (ego-mediated)

According to Freud’s theory of personality, the id is the primal, unconscious part of the psyche that is motivated by the pleasure principle. Seeking immediate gratification of basic desires, it operates without regard for consequences, morality, or social norms. Id-level impulses are instinctive, impulsive, and self-centered. In classical Freudian theory, however, the id does not execute socially organized behavior directly. Rather, it supplies motivational energy that is mediated and structured by the ego in order to function within reality. Accordingly, the examples below are not attributed to the id in isolation, but are interpreted as ego-mediated realizations of underlying id-driven impulses. They may include survival-related and pleasure-seeking activities whose motivational origin can be traced to basic drives such as hunger, comfort, or gratification:

- (e.g., *make* a cup of tea/ coffee/, a sandwich, an omelette, *make* breakfast/ lunch/ dinner, *make* the bed, *make* love, etc.),

pleasure-seeking activities:

- (e.g., *make* friends, *make* a date, *make* my day, *make* fun of, *make* [no] sense, *make* money, *make* a profit, *make* a success of, *make* a demand, *make* a fortune, *make* a living, *make* a loss, *make* a bet, *make* a reservation/ a booking, an arrangement, etc.),

child-like actions

- (e.g., *make* a noise/ a sound; *make* a mess (of), etc.),

peaceful/aggressive actions

- (e.g., *make* peace, *make* war, *make* a killing, *make* a fuss, etc.),

self-centered actions that can be twisted/ fabricated, etc.

- (e.g., *make* the right/wrong choice, *make* up your mind, *make* a complaint, a decision, a joke, a promise, a suggestion, an offer, *make* a choice, a good impression, *make* an effort, *make* an excuse, *make* plans, an arrangement, *make* progress, *make* a comment, *make* a prediction, *make* a difference, *make* an attempt, *make* an exception, *make* a confession, *make* sure, *make* [a/no] difference, *make* a discovery, *make* time for, *make* a move, *make* a pass at, *make* a point, *make* an appearance, *make* an observation, *make* myself clear/ *make* my meaning clear, *make* off with, *make* out [with], *make* up [with], *make* it up, *make* up for [lost time], etc.).

## Discussion

To explore the nuances of verbal usage in English, consider these two examples:

1. If you do not know the answer, then just *‘make’* one up!
2. I really believed my son’s story about the broken window, but he had just *‘made’* it up.

According to the Cambridge Advanced Learner’s Dictionary, ‘*make* [something] up’ is to invent something, such as an excuse or a story, often in order to deceive. The question that arises here is: why is it ‘*make* up’?

Given the proposed analytical framework here, the straightforward answer is that it has to do with deception, which is, in Freud’s terms, motivated by the id as a counterintuitive force of adhering to ethical/moral standards that are calls of the superego. To illustrate, when someone finds themselves in a situation where they have to choose between being ethical (not deceiving) and avoiding punishment or embarrassment (saving face), the ego has to interject to weigh both options given the realities of the situation. The question arises here as to how (and when) the primal instinctual desire wins the conflict. This requires that we consider how deception, as a complex behavior mainly inherent in human interaction that has garnered philosophers, sociologists, and psychologists’ attention, takes place in one’s mind.

Apart from the proposals suggested in all previous models of analysis, we limit our search to the mechanism that Freud’s theory of personality provides. Although forms of deception have been documented in non-human animals, the present discussion focuses on the psychodynamic structure of human deception. Rather than being a pure expression of the id, human deception is better understood as a compromise formation in which id impulses are mediated by ego strategy and constrained by superego regulation. As for the machinery, it results from the ego after coordinating the instinctual urges of the id and the moral requirements of the superego. In simple terms, it is a behavior that originates in the mind due to the complex and conflicting interaction among the id, ego, and superego. The psychological motivation behind deception is a conflict resolution mechanism whose ultimate goal is to seek gratification. It works like this: the desire to deceive originates in the id, to which satisfying instinctual desire outranks fulfilling societal norms or ethical considerations that are normally affected by the influence of the superego. In this case, two conflicting forces compete: the inner impulses of the id and the external reality that calls for satisfying societal norms and ethical considerations pushed by the superego. The ego interjects here to weigh both options and resolve the conflict in the most optimal way possible (given the adversities of the reality, of course). The ego takes the final decision after assessing the feasibility of satisfying the desires of the id and simultaneously managing the consequences of the action in the real world. It is therefore a cost–benefit trade-off. Functioning as an operator in this conflict, the ego is a force that weighs the strategic and pragmatic repercussions of which is costlier and less rewarding: suppressing the urge of the id (by telling the truth to confirm to ethical considerations and societal norms) or fabricating a story (to evade embarrassment, punishment, etc.). Given the uniqueness of each event, the ego takes a calculated decision on either-or basis. What this means is that the decision to deceive is not purely an instinctual act that a person can take unconditionally. As the conflicting demands are ranked based on the ranking of the constraints that regulate them, the ego might sanction deception for either protecting one’s feelings or preventing being harmed. Only when the cost of deception is less than that of tolerating the feelings of guilt or shame can it be sanctioned, and the internal moral disapproval is suppressed. In light of this, deception should be looked at as a psychological process resulting from the complex interaction between the three components of the personality. Given the multifaceted nature of the personality, deception originating in the id is never considered a merely behavioral act. If the superego tolerates deception in light of the adversities of reality, the ego takes the decision into account and decides to sanction it too. Rather than claiming that deception automatically triggers the verb ‘*make’*, the present analysis proposes that the expression ‘*make up’* aligns with the semantic profile of ‘*make’* as an act of construction or fabrication. Deception involves the deliberate creation of a narrative that did not previously exist; it is linguistically conceptualized as bringing something into being rather than performing a routine task. In cognitive-semantic terms, ‘*make’* frequently encodes acts of production, invention, or fabrication, whereas ‘*do’* tends to profile procedural or task-oriented activity. From a psychodynamic perspective, the fabricated narrative serves id-related aims such as self-protection or gratification, but its linguistic encoding reflects how speakers conceptualize deception as an act of creation. The choice of ‘*make’* is therefore motivated by the semantics of fabrication rather than by a direct one-to-one mapping between the id and a verb form. This pattern is consistent with a broader class of expressions in which ‘*make’* profiles acts of conceptual creation or transformation, such as the following:

- (e.g., *make* a claim, *make* money, *make* progress, *make* a difference, *make* a mistake, *make* a decision, *make* a point, *make* a plan, *make* a difference, *make* a change, *make* a move, *make* a difference, *make* a living, *make* a statement, *make* a request, *make* a purchase, *make* a reservation, *make* a list, etc.)

## Superego-driven actions/activities

According to Freud’s theory, the superego is the internalized component of the personality that acts as a conscience that is sanctioned by societal values (Freud, 1923). The component strives to enforce ethical behavior in accordance with societal norms. Therefore, when individuals act in ways that violate their moral principles, the superego causes them to develop a feeling of guilt or shame. Superego-driven actions are, therefore, ethical, self-critical, and idealistic. Actions/ behaviors that are triggered by the superego include: feeling of guilt or shame, adhering to ethical/moral standards, aspiring to achieve the ideal self, and sacrificing for the welfare of others (Freud, 1923; Laplanche and Pontalis, 2018).

## Discussion

The verb ‘*do’* is highly versatile in English, appearing in numerous fixed expressions and idiomatic constructions. Consider the examples below:

- (e.g., Can you *do* me a favor, please?, *do* the dirty on*, do* up [your shoelaces/seatbelt], *do* your hair, *do* your nails, *do* nothing, *do* wrong, *do* a favor, *do* a service, *do* a good deed, *do* harm, *do* badly [in], *do* well, etc.)

These constructions are relevant not only as lexical facts but as indicators of how speakers conceptualize action. Cognitive linguistics holds that recurring linguistic patterns reflect habitual models of experience (Lakoff, 1987; Croft and Cruse, 2004). Expressions built around ‘*do’* tend to encode procedural, duty-oriented, and socially regulated activity. From a psychodynamic perspective, such patterns resonate with ego and superego functions concerned with regulation and responsibility (Freud, 1923). The linguistic pattern therefore provides a conceptual bridge between routine action and psychological structures of control and obligation.

Such actions or behaviors are altruistic and are thus internalized or enforced. During childhood, especially if the child grows up in an environment that stresses religious values and devotion to the welfare of others (Freud, 1923; Ochs and Schieffelin, 1984; Sinclair, 1991; Tomasello, 2009). Self-satisfaction is achieved only if the behavior aligns with societal norms and or ethical personal demands despite enduring hardship (Damon, 1984; Eisenberg, 2000). However, altruistic actions do not go unnoticed; rather, they run counter to the id’s selfish impulses (Freud, 1923). It is only when the superego’s demand to act selflessly overrides the id’s selfish impulses that altruistic behaviors are actualized. The ego, as a mediator, may choose to prioritize self-sacrifice as the best rational course of action by setting aside, at least temporarily, personal desires and by enduring the hardships of taking this course of action (Freud, 1923). For instance, when the child has to choose between eating a piece of chocolate or giving it to her younger sister, the id might push for immediate gratification, and the superego might choose to internalize values of altruism and compassion. At this point, the ego interjects by weighing the consequences. Self-sacrifice would then be more like a responsibility as it is attained after the ego navigates the tension between the selfish drives of the id and the moral imperatives of the superego (Freud, 1923).

The present discussion does not claim corpus-based generalization about the frequency or distribution of these expressions. Rather, the examples are treated as illustrative conceptual cases within a theoretical framework linking psychodynamic interpretation and collocational meaning. A full empirical investigation using large-scale corpora such as COCA or the BNC would be necessary to test distributional patterns quantitatively. The current study is exploratory in nature and aims to propose an interpretive model rather than establish statistical linguistic claims.

### Ego-motivated actions/activities

It has been widely argued that by mediating between the id’s desires and the superego’s moral considerations, the ego strives to find optimal ways to fulfill the id’s demands without severely violating societal norms or causing harm (Freud, 1923). However, not all actions originate in the id (i.e., not all actions are self-centered and/or pleasure-driven). Some actions are reality-driven, task-oriented, etc. As outlined in Freud’s structural theory of personality, the ego is the rational part of the psyche, only constrained by the reality principle. Ego-driven actions are, therefore, rational, practical, and reality-based. These include planning, problem-solving, self-control, and decision-making. Consider the following examples:

- (e.g., *do* the cleaning, do the dishes/ the washing up, *do* the ironing, *do* the laundry, *do* the dusting, *do* housework, *do* your chores, *do* the shopping, *do* homework, *do* an assignment, *do* something, *do* your best/ what you can, *do* your make up, *do* a course, *do* a degree/ an undergraduate degree, *do* a postgraduate degree, *do* a PhD/ a doctorate, *do* an MA, *do* an MBA, *do* research/ some research, *do* a survey, *do* a study, *do* a presentation, *do* a test, *do* a search, *do* a terrible job, *do* a great job, *do* well [in], *do* a bad job, *do* a good job, *do* the right/wrong thing, *do* martial arts, *do* aerobics, *do* the hoovering, *do* a workout, *do* weight training, *do* some work on, *do* the minimum/maximum, *do* business with, *do*… for a living, *do* the accounts/ the books, *do* without, *do* away with, *do* over, *do* up, *do* research, *do* homework, *do* business, *do* exercise, *do* damage, *do* justice, *do* judo, *do* karate, *do* overtime, *do* coffee, etc.)

Given this distinction between actions propelled by the id and those sanctioned by the ego, we can now appreciate the difference between ‘*make coffee’* vs. ‘*do coffee’*. This distinction is not meant to imply a direct causal mapping between grammar and psychic structure. Rather, the argument assumes that linguistic choices reflect recurring conceptual schemas through which speakers interpret action (Lakoff, 1987; Croft and Cruse, 2004). The verbs ‘*make’* and ‘*do’* encode different action models: ‘*make’* profiles acts of creation or experiential engagement, whereas ‘*do’* profiles task execution and obligation. The psychodynamic interpretation is therefore heuristic: it suggests that these conceptual contrasts align with motivational patterns associated with id gratification and ego regulation, without claiming that verb choice is determined by psychology in a literal sense. Whereas the former is an activity where pleasure is sought (e.g., coffee hour as an informal meeting for serving refreshments and chatting), the latter is a formal task that has to be done, i.e., an assigned piece of work is sometimes hard or unpleasant, often requires to be finished within a certain time, etc. It is therefore unnatural to designate a specific future time (e.g., tomorrow at 10:30) for making coffee, but it is natural to do so for ‘*doing coffee’*. For this, the lists of ‘*do’* and ‘*don”t* are often seen hanging all over the walls of workplaces.

### ‘Doing’ vs. ‘making’: linguistic framing and legal responsibility

The relevance of this section lies in how legal and everyday discourse distinguish between actions described with ‘*do’* and those described with ‘*make’*. Legal language frequently encodes agency, responsibility, and intentionality through verb choice. Expressions such as ‘*do harm’*, ‘*do wrong’*, or ‘*do damage’* frame actions as accountable deeds, whereas constructions with ‘*make’* often highlight creation, fabrication, or causation (e.g., *make* a threat, *make* a false statement). The following discussion uses Freud’s framework to interpret how these linguistic distinctions mirror different conceptualizations of agency and responsibility in legal reasoning.

Sigmund Freud’s theory of personality, which consists of three components (the id, ego, and superego), offers a compelling framework for understanding human behaviors/actions, etc., along these lines:

- Self-centered—id
- Self-control—ego
- Self-critical—superego

The theory underlies intriguing challenges when considering legal implications and justice. Hence, each component of the personality plays a role in how individuals make decisions, including those that lead to actions with legal consequences. Let us break this down by looking at each aspect and its potential legal repercussions.

### Legal repercussions of id-driven actions

Actions such as theft or violence are often interpreted as involving impulses toward immediate gratification (Freud, 1923); however, empirical research shows that many such acts also involve planning and strategic reasoning. From a psychodynamic perspective, these behaviors are better understood as outcomes of interaction between id impulses and ego organization rather than as purely id-driven actions. Legal systems accordingly evaluate such acts in relation to responsibility and intent (Morse, 2004; Shute et al., 1993). The law holds individuals accountable for their inner urges because such actions could harm others. In the eyes of the law, people taking these actions should face legal punishment, though lawyers might argue for dismissing responsibility for id-driven impulses, claiming that they occur when the individual lacks control over his/her actions. The law does not entirely absolve individuals of responsibility because they just acted impulsively or in the heat of passion. It is only when such actions are executed due to serve mental illness that the doer might be found not guilty because of insanity. However, this should be a rare exception as it involves significant legal and psychological assessments. Within the theoretical distinction proposed in this study, ‘*make’* is associated with actions conceptually framed as originating in impulse, creation, or self-directed drive, whereas ‘*do’* is associated with procedural or reality-managed action. From that interpretive perspective, an id-propelled crime can be read as an act that is linguistically conceptualized as ‘*made*’ rather than ‘*done*’. This claim is offered as a theoretical interpretation within our framework rather than as a categorical rule of English usage.

### Legal repercussions of ego-driven actions

Individuals are fully held accountable when their actions are triggered by the ego; hence, such actions are often viewed as thoughtful, calculated, and guided by awareness of consequences. Criminal law traditionally places strong emphasis on intentional action and rational decision-making when assigning culpability, particularly through doctrines related to *mens rea*, premeditation, and conscious risk assessment (Hart, 2008; Morse, 2004). More interestingly, the legal system expects individuals to balance personal desires with societal expectations—a task that parallels Freud’s characterization of the ego as a mediator governed by the reality principle. When someone commits a crime while fully aware of the consequences, the law treats the actor as fully responsible because the behavior reflects deliberation rather than uncontrollable impulse (Duff, 2018; Slobogin, 2006). Actions such as fraud, embezzlement, and financial manipulation fall into this category, as they typically involve planning, foresight, and calculated benefit–risk evaluation. In such cases, courts are generally reluctant to grant leniency based on claims of diminished control, since the behavior demonstrates cognitive organization and purposeful intent (Morse, 2004). From a psychodynamic perspective, these are actions in which the ego has successfully coordinated internal drives with external reality, resulting in behavior that is socially legible as intentional wrong doing. Expressions such as ‘X did it*’* may be read illustratively as everyday ways of attributing agency and responsibility; however, this example is intended as a conceptual metaphor within our theoretical framework rather than as empirical linguistic evidence linking ‘*do’* exclusively to ego-driven action.

### Legal repercussions of superego-driven actions

If an individual breaks the law but experiences guilt or remorse afterward, legal systems may take such psychological states into account during sentencing, particularly in discussions of mitigation, moral blameworthiness, and character evaluation (Hart, 2008; Duff, 2018; Tadros, 2007). Criminal jurisprudence has long recognized that remorse, intent, and moral awareness influence judicial assessments of culpability, even when legal guilt is established (Morse, 2004; Slobogin, 2006; Robinson, 1997). Courts frequently consider emotional disturbance, provocation, and moral conflict when distinguishing between degrees of homicide or when determining partial defenses and sentencing mitigation (Ashworth and Horder, 2013). Actions such as manslaughter, excessive self-defense, or crimes committed under extreme emotional stress are evaluated within legal frameworks that explicitly address diminished responsibility and moral complexity (Robinson, 1997; Morse, 2004). Some actions may be interpreted as attempts to reconcile competing duties, for instance, protecting others while violating legal norms, and such cases are occasionally treated as grounds for partial justification or excuse (Ashworth and Horder, 2013; Duff, 2018). From a psychodynamic perspective, these cases may be read as reflecting the operation of the superego, where guilt, moral judgment, and internalized ethical standards shape behavior and its interpretation. This discussion does not claim a direct legal mapping of Freudian categories, but proposes an interpretive lens through which established legal reasoning about remorse and responsibility can be conceptually understood.

## Conclusion

The distinction between the verbs ‘*make’* and ‘*do’* may reveal something deeply significant about how we think, feel, and interpret the world around us. When we look at these verbs through Freud’s theory of personality, they appear to be more than habitual usage. They may express our desires, our sense of responsibility, and our moral instincts. This may help explain why native English speakers use ‘*make’* and ‘*do’* with ease, while learners of English find them confusing or inconsistent. The difficulty is not a matter of intelligence, but rather the extent to which language is intertwined with emotional and psychological patterns that are rarely addressed in grammar instruction.

By linking language to individuals’ psychological components, this research offers a new way to understand how language is used. The research suggests that English language instruction should go beyond simply explaining differences in meaning based on grammar rules and should incorporate psychological and conceptual motivations behind word choice, a need also reflected in recent pedagogical research in translation studies (Algazo et al., 2025; Al-Shahwan, 2024). The significance of this study lies in its novel approach: it introduces a new perspective on language by linking everyday word choice to Freud’s structural model of the psyche. This interdisciplinary connection offers a fresh theoretical lens through which to interpret linguistic behavior, potentially opening new pathways for future research. Scholars in both linguistics and psychology may build upon this foundation to further investigate how specific lexical choices reflect underlying psychological structures such as the id, ego, and superego. By integrating psychoanalytic theory with linguistic analysis, this research broadens the scope of how language can be studied, understood, and taught.

Future research could examine other verbs in English, expanding the hypothesis that verb choice is linked to individual psychological structures and enhancing our understanding of how psychological components influence linguistic usage. It is also recommended that this research be extended to include other languages, so we can better understand how word choice relates to speakers’ psychological components. Taking a closer look at how both native and non-native speakers perceive and internalize these verbs could give us deeper insight into the psychological factors that influence how people use and learn language.

## Data Availability

The original contributions presented in the study are included in the article/supplementary material, further inquiries can be directed to the corresponding author.

## References

[ref1] Abu Helal A. R. (2023). On the semantics of (negated) approximative kaada in classical Arabic: a case for embedded exhaustification. Linguistics Vanguard 9, 37–49. doi: 10.1515/lingvan-2022-0106

[ref2] Aitchison J. (2000). The Seeds of speech: Language origin and Evolution. Cambridge: Cambridge University Press.

[ref3] Algazo M. Clark J. B. Swaie M. Alghazo S. (2025). Exploring the factors driving L1 use in Jordanian secondary EFL classrooms. Cogent Education 12, 1–17. doi: 10.1080/2331186X.2025.2560055

[ref4] Allwood J. S. Gärdenfors P. eds. (1999). Cognitive Semantics: Meaning and Cognition (Vol. 55). Amsterdam: John Benjamins Publishing doi: 10.1075/pbns.55

[ref1001] Almegren A. (2022). Effect of corpus-based activities on learning verb–noun collocations in Saudi EFL classes. Jordan Journal of Modern Languages and Literatures 14, 371–383. doi: 10.47012/jjmll.14.2.8

[ref5] Al-Shahwan R. (2024). The attitudes of students at the translation Department at Al-Zaytoonah University of Jordan towards using translation from English language into Arabic language by the instructors as a medium of instruction in translation courses. Al-Zaytoonah University of Jordan Journal for Human & Social Studies 5, 176–190. doi: 10.15849/zjjhss

[ref6] Anderson J. (2025). Common Grammatical Challenges for Arabic Language Learners: A Comparative Study of errors among Multilingual Speakers. (Manuscript in preparation). Retrieved May 15, 2025 from https://www.researchgate.net/publication/388190045

[ref7] Ashworth A. Horder J. (2013). Principles of criminal law. Oxford: Oxford University Press doi: 10.1093/he/9780199672684.001.0001

[ref8] Bechtel W. (2001). “*Linking cognition and brain: the cognitive neuroscience of language*,” in Philosophy and the Neurosciences: A reader, eds. Bechtel W. Mandik P. Mundale J. Stufflebeam R. (Malden: Blackwell Publishers), 152–171.

[ref9] Bickerton D. (1990). Language and Species. Chicago: University of Chicago Press doi: 10.7208/chicago/9780226220949.001.0001

[ref10] Bloomfield L. (1933). Language. New York: Henry Holt and Company.

[ref11] Busch F. Delgado N. (Eds.). (2024). The ego and the id: 100 Years later. London: Routledge doi: 10.4324/9781003336754

[ref12] Bybee J. (2010). Language, Usage and Cognition. Cambridge: Cambridge University Press doi: 10.1017/CBO9780511750526.

[ref14] Chomsky N. (1965). Aspects of the Theory of Syntax. Cambridge: M.I.T. Press.

[ref15] Chomsky N. (1975). Reflections on Language. New York: Pantheon Books.

[ref16] Christiansen M. H. Chater N. (2008). Language as shaped by the brain. Behav. Brain Sci. 31, 489–509. doi: 10.1017/S0140525X08004998 18826669

[ref17] Croft W. Cruse D. A. (2004). Cognitive Linguistics. Cambridge: Cambridge University Press doi: 10.1017/CBO9780511803864

[ref18] Cruse D. A. (2000). Meaning in Language: an Introduction to Semantics and Pragmatics. Oxford: Oxford University Press.

[ref19] Dajani B. A. S. Mubaideen S. Omari F. M. A. (2014). Difficulties of learning Arabic for non-native speakers. Procedia Soc. Behav. Sci. 114, 919–926. doi: 10.1016/j.sbspro.2013.12.808

[ref20] Damon W. (1984). “*Self-understanding and moral development from childhood to adolescence*,” in Moral development, ed. Damon W. (San Francisco: Jossey-Bass), 109–127.

[ref21] Duff R. A. (2018). The Realm of criminal law. Oxford: Oxford University Press doi: 10.1093/oso/9780199570195.001.0001

[ref22] Eco U. (1997). The search for the perfect Language. Oxford: Blackwell.

[ref23] Eisenberg N. (2000). Emotion, regulation, and moral development. Annu. Rev. Psychol. 51, 665–697. doi: 10.1146/annurev.psych.51.1.665 10751984

[ref24] Ellis N. C. (2005). At the interface: dynamic interactions of explicit and implicit language knowledge. Stud. Second. Lang. Acquis. 27, 305–352. doi: 10.1017/s027226310505014x

[ref25] Evans V. (2009). How Words mean: Lexical Concepts, Cognitive Models, and Meaning Construction. Oxford: Oxford University Press doi: 10.1093/acprof:oso/9780199234660.001.0001

[ref26] Evans V. Green M. (2018). Cognitive Linguistics: An Introduction. London: Routledge doi: 10.4324/9781315864327

[ref28] Freud S. (1923). *The ego and the id*. In Strachey J. (Ed. & Trans.), The Standard Edition of the Complete Psychological Works of Sigmund Freud (Vol. 19, pp. 1–66). London: Hogarth Press.

[ref2008] Gardner H. (1985). The Mind’s New Science: A History of the Cognitive Revolution. New York: Basic Books.

[ref29] Geeraerts D. (2010). Theories of lexical Semantics. Oxford: Oxford University Press.

[ref30] Goldberg A. E. (2006). Constructions at work: The Nature of Generalization in Language. Oxford: Oxford University Press.

[ref31] Grammarly. (n.d.). Do vs. make: What’s the Difference? Retrieved January 22, 2025 from https://www.grammarly.com/commonly-confused-words/do-vs-make

[ref32] Gries S. T. (2013). Statistics for Linguistics with R: A Practical Introduction. 2nd Edn Berlin: De Gruyter Mouton doi: 10.1515/9783110307474

[ref33] Harnad S. (1990). The symbol grounding problem. Physica D: Nonlinear Phenomena 42, 335–346. doi: 10.1016/0167-2789(90)90087-6

[ref34] Hart H. L. A. (2008). Punishment and Responsibility: Essays in the Philosophy of law. Oxford: Oxford University Press doi: 10.1093/acprof:oso/9780199534777.001.0001

[ref35] Lakoff G. (1987). “*What categories reveal about the mind*,” in Women, fire, and Dangerous Things: What Categories reveal about the mind, ed. Lakoff G. (Chicago: University of Chicago Press), 5–46. doi: 10.7208/chicago/9780226471013.001.0001

[ref36] Lakoff G. Johnson M. (1999). Philosophy in the Flesh: The Cognitive Unconscious and the Embodied mind: How the Embodied mind Creates Philosophy. New York: Basic Books.

[ref37] Langacker R. W. (1987). Foundations of Cognitive Grammar. Stanford: Stanford University Press.

[ref38] Langacker R. W. (2008). Cognitive Grammar: A basic Introduction. Oxford: Oxford University Press doi: 10.1093/acprof:oso/9780195331967.001.0001

[ref39] Laplanche J. Pontalis J. B. (2018). The Language of Psychoanalysis. London: Routledge.

[ref40] Lewis M. (2000). Teaching Collocation: Further Developments in the Lexical Approach. Hove: Language Teaching Publications.

[ref41] Lyons J. (1995). Linguistic Semantics: an Introduction. Cambridge: Cambridge University Press doi: 10.1017/CBO9780511810213

[ref42] Mace J. (1999). Arabic Verbs and Essential Grammar. Lincolnwood: NTC Publishing.

[ref43] Morse S. J. (2004). Reason, results, and criminal responsibility. Univ. Ill. Law Rev. 2004, 363–444.

[ref44] Murphy M. L. (2003). Semantic Relations and the Lexicon: Antonymy, Synonymy and other Paradigms. Cambridge: Cambridge University Press doi: 10.1017/CBO9780511486494.

[ref45] Murphy R. (2019). English Grammar in Use. 5th Edn Cambridge: Cambridge University Press.

[ref46] Nesselhauf N. (2005). Collocations in a Learner corpus: Studies in corpus Linguistics, vol. 14 Amsterdam: John Benjamins Publishing doi: 10.1075/scl.14

[ref47] Ochs E. Schieffelin B. (1984). “Language acquisition and socialization: three developmental stories,” in Culture Theory: Essays on mind, Self and Emotion, eds. Shweder R. A. LeVine R. A. (Cambridge: Cambridge University Press), 276–320.

[ref48] Perfect English Grammar. (n.d.). Make or do. Retrieved January 22, 2025 from https://www.perfect-english-grammar.com/make-or-do.html

[ref49] Pinker S. (2007). *The Language instinct: how the mind Creates Language* (1st Harper Perennial Modern Classics ed.). New York: Harper Perennial Modern Classics.

[ref50] Ricœur P. (1970). Freud and Philosophy: an Essay on Interpretation. New Haven: Yale University Press.

[ref51] Robinson P. H. (1997). Structure and Function in criminal law. Oxford: Oxford University Press doi: 10.1093/acprof:oso/9780198258865.001.0001

[ref52] Searle J. R. (1980). Minds, brains, and programs. Behav. Brain Sci. 3, 417–424. doi: 10.1017/S0140525X00005756

[ref53] Shute S. Gardner J. Horder J. (Eds.). (1993). Action and Value in criminal law. Oxford: Oxford University Press doi: 10.1093/acprof:oso/9780198258063.001.0001

[ref54] Sinclair J. (1991). Corpus, concordance, collocation. Oxford: Oxford University Press.

[ref55] Skinner B. F. (1957). Verbal behavior. New York: Appleton-Century-Crofts doi: 10.1037/11256-000

[ref56] Slobogin C. (2006). Proving the Unprovable: The role of law, Science, and Speculation in Adjudicating Culpability and Dangerousness. Oxford: Oxford University Press doi: 10.1093/acprof:oso/9780195189957.001.0001

[ref57] Stanojević M. (2009). Cognitive synonymy: a general overview. Facta Universitatis, Series: Linguistics and Literature 7, 193–200.

[ref58] Stefanowitsch A. Gries S. T. (2003). Collostructions: investigating the interaction of words and constructions. International journal of corpus linguistics 8, 209–243. doi: 10.1075/ijcl.8.2.03ste

[ref59] Swan M. (2005). Practical English Usage 3rd edition. Oxford: Oxford university press.

[ref60] Tadros V. (2007). Criminal responsibility. Oxford: Oxford University Press doi: 10.1093/acprof:oso/9780199225828.001.0001

[ref61] Tomasello M. (2009). Why we Cooperate. Cambridge: MIT Press doi: 10.7551/mitpress/8470.001.0001

